# The Impact of Corporate Social Responsibility Performance Feedback on Corporate Social Responsibility Performance

**DOI:** 10.3389/fpsyg.2022.893193

**Published:** 2022-05-18

**Authors:** Jae-Eun Lee, Young Soo Yang

**Affiliations:** ^1^Department of International Trade, Sunchon National University, Suncheon, South Korea; ^2^Department of Global Business, Hanshin University, Osan, South Korea

**Keywords:** performance feedback, aspiration, corporate social performance, behavioral theory of the firm (BTOF), CSR

## Abstract

This study empirically analyzes how corporate social responsibility (CSR) performance feedback impacts CSR performance, focusing on the performance feedback perspective of behavioral theory of the firm (BTOF). By performing generalized least squares (GLS) regression analysis based on Korean company data from 2012 to 2019, we presented evidence that positive social and historical performance feedback had a positive effect on CSR performance. Our results provide evidence that firms with higher social and historical CSR performance than CSR aspiration may have higher CSR performance than those that do not.

## Introduction

Corporate social responsibility (CSR) can be defined as a firm’s core strategy for voluntarily reflecting social and environmental concerns in the operation of the business to interact with various stakeholders (Wang et al., 2018, p. 68). CSR has attracted scholarly attention, and CSR has increased gradually owing to the growing, recent perception that sustainability is crucial for a firm’s long-term growth and survival (Bahta et al., 2021). Many researchers and managers are prioritizing CSR to create a sustainable competitive advantage (Lee and Lee, 2019; Kim and Kim, 2020; Matten and Moon, 2020). However, the degree and pattern of CSR activities performed by firms vary greatly among firms, and CSR performance due to CSR activities also vary greatly from firm to firm. Therefore, many scholars have attempted to identify corporate decisions to participate in CSR activities and determinants of CSR performance (Kim and Kim, 2020; Yuan et al., 2020; Ben-Amar et al., 2021). The previous studies have stressed that firms participate in CSR activities to increase stakeholder value based on the stakeholder theory, arguing that firm CSR performance is eventually related to the financial firm performance (Hillman and Keim, 2001; Kim et al., 2019). Conversely, based on the trade-off theory, other scholars emphasize that CSR activities negatively affect financial performance as they force firms to spend unnecessary money and eventually worsen profitability (McWilliams and Siegel, 2001; Moore, 2001; López et al., 2007).

Meanwhile, many scholars in the field of organizational theory and strategic management have used the performance feedback perspective based on the behavioral theory of the firm (hereafter, BTOF), which has been presented as a major theoretical basis for explaining corporate performance (Cyert and March, 1963). From the BTOF aspect, the difference between a firm’s actual performance and aspiration level of performance, that is, attainment discrepancy, affects firm strategy or behavior (Cyert and March, 1963). Attainment discrepancy is crucial in the performance feedback model of the BTOF. Moreover, the performance feedback model is considered to evaluate one’s performance based on the level of performance that firms aspire to Cyert and March (1963) and Alessandri and Pattit (2014). Essentially, based on the actual firm performance compared and evaluated based on the performance of the aspiration level, the CEO determines the effectiveness of the current firm strategy (Audia and Greve, 2006; Lu and Wong, 2019). Applying these discussions to the CSR context, the strategic behaviors that firms can choose may vary depending on whether CSR performance is high or low compared to aspiration level. However, previous studies that have sufficiently discussed this are scarce. Additionally, the difference between CSR aspiration level and actual CSR performance, that is, the effect of attainment discrepancy on CSR performance, remains unclear.

As Nason et al. (2018) highlighted the discussion on social performance feedback compared to financial performance feedback is lacking and attempts to examine how a firm’s strategic behavior changes according to corporate non-financial social performance feedback have been relatively insufficient. Few studies have attempted to apply the performance feedback perspective of BTOF to the CSR context (Arora and Dharwadkar, 2011). For example, Arora and Dharwadkar (2011) found that attainment discrepancy moderates the relationship between corporate governance and CSR based on the BTOF perspective. However, in the study of Arora and Dharwadkar (2011), since attainment discrepancy was measured based on the financial performance, there is a big difference from our study that measures attainment discrepancy based on the CSR performance. Additionally, Xu and Zeng (2020) are similar to this study in that it investigates the relationship between CSR’s attainment discrepancy and CSR performance, not financial performance. However, their study considers only the social corporate social performance (CSP) aspiration level, while ours study includes both social and historical CSP aspiration levels.

To fill this research gap, this study investigates the impact of CSR performance feedback on future CSR performance. We present the following research questions. First, how does positive CSR performance feedback (positive attainment discrepancy wherein CSR performance exceeds CSR aspiration levels) affect future CSR performance? Second, how does negative CSR performance feedback (negative attainment discrepancy in which CSR performance is below CSR aspiration levels) affect future CSR performance?

## Theoretical Background and Hypothesis Development

### Performance Feedback and Corporate Social Responsibility Performance

One of the main theories explaining firm CSR performance is the perspective of performance feedback of BTOF. In the BTOF perspective, organizations form levels of aspiration for their goals and choose courses of their actions that can help them to achieve that level of aspiration (Cyert and March, 1963; Kotiloglu et al., 2020). The BTOF emphasizes organizational processes such as performance evaluation, search, and decision-making (Cyert and March, 1963; Greve, 2003). Considering performance feedback from the BTOF, as organizations are considered a goal-directed system (Chen and Miller, 2007) using simple decision-making rules to change their activities, the firm will evaluate their performance based on aspiration levels and respond differently depending on whether performance is higher or lower (Cyert and March, 1963; Greve, 2003). According to the perspective of performance feedback, aspiration levels are the reference point for evaluating the organizational performance (Kotiloglu et al., 2020). In other words, the aspiration level becomes the criterion for decision makers to judge satisfaction and dissatisfaction with their strategic results (Simon, 1955). By evaluating one’s performance using aspiration level as a reference point, organizations that recognize its success or failure can change the direction and scope of organizational search to enhance performance (Greve, 2003). Aspiration level can be divided into the historical and social aspiration levels. The former is formed through past experience organizational experience and latter through comparison with the reference group (Cyert and March, 1963; Greve, 1998). Historical aspiration (HA) level considers the organization’s past experience as a major reference point, mainly by comparing current performance with the organization’s past performance (Greve, 2003). Conversely, the social aspiration level is determined by comparing the performance of the reference group with that of the firm. Studies have emphasized that both historical and social aspiration level should be considered (Bromiley, 1991; Greve, 1998). Hence, this study considers both historical and social aspiration levels. The decision maker determines the type of search, such as problemistic or slack search, through the process of comparing aspiration level and one’s performance. If firm performance does not reach the aspiration level, this signals the decision maker that a problem has occurred in the current organization and prompts a problemistic search that makes efforts to compensate for the current performance that falls short of expectations. In this problemistic search process, decision makers make decisions to take more risks and actively solve problems. Conversely, if a firm’s performance exceeds its aspiration level, decision makers feel no need to change because they see the current situation as profitable and tend to maintain or wait and monitor the current situation (Cyert and March, 1963). When a firm’s performance exceeds its aspiration level, it conducts slack search even if it conducts search, and firms hope that the current situation will be maintained (Cyert and March, 1963; Greve, 2003). We attempt to apply this discussion to the CSR context in this study. In this study, CSR performance feedback was considered a major antecedent factor in CSR performance as decision makers can make different decisions related to CSR depending on whether a firm’s CSR performance is high (positive) or low (negative) compare with their CRS aspiration level.

### Research Hypotheses

In this study, the difference in the CSR performance compared to the CSR aspiration level of a firm (positive or negative attainment discrepancy) influences the firm’s strategy or behaviors related to CSR according to the BTOF’s performance feedback perspective. Particularly, when a firm’s CSR performance is low compared to CSR aspiration level (negative CSR performance feedback), firm decision-makers may recognize low CSR performance as an important problem and conduct problemistic search to improve it (Zhong and Ren, 2021). Here, CSR aspiration level can be divided into historical and social CSR aspiration level. Decision makers with bounded rationality can make appropriate decisions by comparing past and present CSR performance. Hence, this CSR aspiration level can consider the historical CSR aspiration level. Additionally, decision makers with bounded rationality can make CSR-related decisions by comparing the CSR performance of their reference groups with their CSR performance. Hence, the CSR aspiration level simultaneously becomes the social CSR aspiration level. Many previous studies emphasize that negative performance feedback generates the problemistic search (Cyert and March, 1963). The problemistic search can be considered a solution for firms having lower performance relative to aspiration level (Iyer and Miller, 2008; Posen et al., 2018; Choi J. et al., 2019). In addition, performance below the aspiration level tends to cause firms to solve problems faced by encouraging more innovative activities (Lu and Wong, 2019). If the CSR performance is low as compared to the CSR aspiration levels, firms may be threatened with legitimacy for CSR, and the need to secure legitimate CSR for sustainable growth and survival increases (DiMaggio and Powell, 1983; Du and Vieira, 2012). Receiving negative performance feedback may reduce external stakeholders’ trust in the corporate decision makers. Additionally, receiving negative feedback can further strengthen external pressure requiring stakeholders to achieve their goals, limiting management autonomy (Arora and Dharwadkar, 2011). Therefore, receiving negative CSR performance feedback may motivate firms to spend more CSR costs to minimize the negative impact and restore legitimacy and trust from stakeholders. In the end, the negative CSR performance feedback could have a positive effect on CSR performance (Park, 2007; Kim et al., 2015; Xu and Zeng, 2020). Hence, the following hypothesis was derived.

*H1*: Negative CSR performance feedback (in that as CSR performance falls below CSR aspiration level) is positively related to CSR performance.

Conversely, according to the performance feedback perspective of BTOF, when a firm’s financial performance exceeds aspiration level, decision-makers feel no need to change because they see the current situation as profits (Lu and Fang, 2013). As firms have already achieved their high level of financial performance they aspire to, decision-makers are unaware of the need for additional search to take additional risks and further enhance financial performance (Audia et al., 2000). Managers do not perceive their financial performance as a problem if their financial performance exceeds their aspiration level. Hence, even if they conduct a search, they will attempt to maintain the current situation by mainly conducting a slack search. These conservative tendencies of decision-makers have been confirmed in many previous studies. For example, Greve (1998) emphasized that as a result of conducting an empirical analysis on the US radio industry, if firm performance is higher than the aspiration level, the probability of strategic change becomes exceedingly lower. Basically, when financial performance is generally higher than the aspiration level, firms will tend to maintain the phenomenon without making additional efforts to improve financial performance (Lucas et al., 2018). However, considering that CSR requires fulfilling a non-financial aspect of a firm, a slightly different discussion is possible in the CSR context. CSR needs to consider a wide range of stakeholders who have relationships with firms as it goes beyond the general responsibility that firms must legally comply with and includes ethical and moral responsibilities. When firm CSR performance is high compared to the CSR aspiration level, stakeholders related to the firm can show trust in firm decision makers. This provides decision makers with more discretion over resource allocation (Arora and Dharwadkar, 2011). If firm decision makers are satisfied with the current situation and reduce the budget or expenditure required for CSR activities, stakeholders may doubt the authenticity of the activities. As CSR is related to the perceptions of various stakeholders, including consumers, even if CSR performance is higher than CSR aspiration levels, firm decision makers can have a positive impact on CSR performance by continuously spending CSR costs to ensure legitimacy without reducing CSR commitment. This argument can also be confirmed in the previous empirical studies. Xu and Zeng (2020) conducted empirical analysis on Japanese companies in anticipation of a negative impact on philanthropic/environmental expenditure if they performed higher than the level of philanthropic/environmental aspirations of firms. However, owing to empirical analysis, and contrary to the authors’ expectations, empirical analysis results were presented wherein positive attainment discrepancy in corporate philanthropic/environmental performance had positive effect on philanthropic/environmental expenditure, respectively. Additionally, when a slack search is performed compared to a problematic search, slack resources will likely be formed because of the additional room for resource utilization. Firms with abundant organizational slack resources can enable more experimentation and organizational change than those that do not (March, 1981). If a firm receives positive CSR performance feedback, it can secure authenticity and legitimacy for CSR activities from stakeholders, including consumers, and can perform more experimental and active CSR activities to strengthen their positive corporate image. Therefore, positive CSR performance feedback could have a positive effect on CSR performance. We propose the following hypothesis:

*H2*: Positive CSR performance feedback (in that as CSR performance rises above CSR aspiration level) is positively related to CSR performance.

## Research Methods

### Sample and Data

In this study, we matched the Korea Economic Justice Research Institute (KEJI) index and firm-level information to those firms selected in the top 200 selected by the KEJI (Oh et al., 2019). Data were collected from archival data sources such as KIS-Value, TS2000, DART as an electronic disclosure system, and KEJI for KEJI index. Our initial sample was obtained from KEJI for 2012 to 2019 and merged these firms with financial data using KIS-Value and TS2000. Our final dataset comprises 1091 observations for 8 years of publicly listed firms on Korea Stock Exchange (KSE) from 2012 to 2019. We employed a 1-year lagged structure between dependent and independent variables and control variables to avoid any reverse causality (Oh et al., 2019).

### Variables and Measurement

#### Dependent Variable

CSR performance was measured in various ways in previous studies, using scores announced by specific organizations (Jung and Kim, 2016; Jeong et al., 2018) or the donation amount or donation ratios (Choi Y. K. et al., 2019; Wang et al., 2021). Following earlier studies (Jung and Kim, 2016; Chang et al., 2017; Jeong et al., 2018; Oh et al., 2019), as the proxy for the social corporate responsibility performance for this study, we used the KEJI index, which is announced annually. The KEJI index is one of the representative CSR performance indicators used in Korean studies (Oh et al., 2019) and is similar to the index such as Kinder, Lydenberg which evaluate the CSR index of S&P 500 (Jung and Kim, 2016).

Since 1991, the KEJI has developed its own evaluation model. CSR performance is quantitatively calculated using accounting information data for KOSPI-listed companies. KEJI has evaluated various aspects and characteristics for selecting the 200 largest companies in Korea (Jung and Kim, 2016). The KEJI index consists of six criteria with a total score of 100 points: soundness (25 points), fairness (20 points), contribution to social service (15 points), consumer protection satisfaction (15 points), environmental protection satisfaction (10 points), and employee satisfaction (15 points). Until 2011, contribution to economic development was included in the KEJI index. However, in 2012, the corresponding item was removed from the index. This study measured the CSR performance as the dependent variable by using the total score of KEJI (Jung and Kim, 2016; Jeong et al., 2018; Oh et al., 2019).

#### Independent Variables

Corporate social responsibility performance feedback: We analyzed CSR performance feedback using CSR performance instead of corporate financial performance to measure corporate CSR performance feedback (Xu and Zeng, 2020; Wang et al., 2021). The amount of donation was widely used to proxy CSR performance in earlier studies (Choi Y. K. et al., 2019; Wang et al., 2021), we used each firm’s donation amount to measure CSR performance aspiration. In this study, performance feedback was classified into two types, HA level and social aspiration level. Both effects were analyzed accordingly (Manzaneque et al., 2020).

Social aspiration (SA) was measured as the average donation expenditure of firms in the same industry except for the focal firm based on two-digit level of the KSIC codes (Greve, 2003; Manzaneque et al., 2020; Xu and Zeng, 2020; Ye et al., 2021). Following previous studies (Manzaneque et al., 2020; Ye et al., 2021), HA was measured by the difference between a firm’s CSR performance and past aspiration level and is measured as each firm’s amount of donation in year t-1.

Based on the previous research on the performance feedback formulas (Xu and Zeng, 2020), we measured the positive CSR performance feedback and negative CSR performance feedback for both social and historical CSR performance feedback.

Positive CSR Performance Feedback_i_
=CSR Performance_i_ − Aspiration_i_ if CSR Performance_i_ > Aspiration_i_
= 0 if CSR Performance_i_ ≤ Aspiration_i_
Negative CSR Performance Feedback_i_
= Aspiration_i_ − CSR Performance_i_ if CSR Performance_i_ < Aspiration_i_
= 0 if CSR Performance_i_ ≥ Aspiration_i_
; where i = focal firm

#### Control Variables

We controlled for firm- and industry-level factors that could affect a CSR. Generally, large-size firms receive most of the social attention, increasing stakeholder pressure, and imposing high expectations for socially responsible behavior (Xu and Zeng, 2020). Therefore, in this study, firm size was included as a control variable, and firm size was measured as a log value of total sales (Attig et al., 2016; Chang et al., 2017; Xu and Zeng, 2020).

The previous studies have reported that firm age has a positive or negative relationship with corporate CSR performance (Chang et al., 2017). Hence, to control for the age of the firm, we included firm age as the control variable by measuring the number of years since the firm establishment (Xu and Zeng, 2020).

The previous studies argued that firm financial status has great influence on the CSR performance (Chang et al., 2017). Therefore, return on asset (ROA), a representative measure of firm financial performance, was included as a control variable (Xu and Zeng, 2020). ROA was measured by dividing net income by total assets (Chang et al., 2017; Jeong et al., 2018).

Slack influences the search behavior of the behavioral theory of the firm (Greve, 2003; Chen, 2008). To control for the effect of potential slack on CSR performance of financial position, the debt-to-equity ratio of the firm was controlled (Chen, 2008).

Tobin’s Q was included as a proxy for corporate value (Jeong et al., 2018; Wang et al., 2021), and Tobin’s Q is a representative proxy for market value, reflecting the firm’s future profits (Cho et al., 2019). Tobin’s Q was calculated as follows.


$${\mathrm{Tobin}}^{\prime }sQ=[(\mathrm{Stock}\mathrm{market}\mathrm{price}\mathrm{per}\mathrm{common}\mathrm{share}{\mathrm{Tobin}}^{\prime }sQ=[(\mathrm{Stock}\mathrm{market}\mathrm{price}\mathrm{per}\mathrm{common}\mathrm{share}\times \mathrm{Number}\mathrm{of}\mathrm{common}\mathrm{shares})+\left(\mathrm{Book}\mathrm{value}\mathrm{of}\mathrm{total}\mathrm{debt}\right)]/\times \mathrm{Number}\mathrm{of}\mathrm{common}\mathrm{shares})+\left(\mathrm{Book}\mathrm{value}\mathrm{of}\mathrm{total}\mathrm{debt}\right)]/\mathrm{Book}\,\mathrm{value}\,\mathrm{of}\,\mathrm{total}\,\mathrm{asset}\mathrm{Book}\,\mathrm{value}\,\mathrm{of}\,\mathrm{total}\,\mathrm{asset}$$

Since the degree of the internationalization of the firm acts as a pressure to improve corporate CSR (Attig et al., 2016; Xu and Zeng, 2020), the degree of internationalization of a firm was included as a control variable. Hence, we measured the level of internationalization by the ratio of foreign to total sales (FSTS), the most representative indicator of the degree of the internationalization (Attig et al., 2016).

In this study, the industry rivalry, the industry annual sales growth rate and the industry dummy were included in the analysis to control for industry differences owing to the different characteristics of each industry (Chen, 2008). To control for the rivalry within an industry, the degree of competition within the industry was measured by a number of firms in each industry according to previous studies (Lee et al., 2009; Ye et al., 2021). The industry dummy was controlled by making each industry a dummy variable based on the three-digit level (middle-level) of the KSIC code.

### Statistical Analysis

Since data for this study are panel data, and both cross-sectional and time series analysis are required. Additionally, because of potential heteroskedasticity and serial correlation problem in panel data, generalized least squares (GLS) regression analysis is suitable for this study (Lee et al., 2009; Wang et al., 2021). The Hausman Test was conducted to select an accurate analysis method. The analysis shows that the random-effect model was considered a more efficient estimating equation than fixed-effect model. Thus, we ran random-effects GLS regression to test our hypotheses.

## Results

Table 1 shows the descriptive statistics and correlation coefficients of our study. To test for multicollinearity, we calculated the variance inflation factor (VIF) (Oh et al., 2019). The result shows that the VIF value was less than 2 in all models, indicating that there is no multicollinearity problem (Chatterjee et al., 2000).

**TABLE 1:** Descriptive statistics and correlations.

	Mean	S.D.	1	2	3	4	5	6	7	8	9	10	11	12	13
1	64.252	2.310	1												
2	25.992	1.776	0.2056	1											
3	38.051	19.743	–0.0548	–0.0273	1										
4	0.015	0.154	0.0379	0.1325	–0.0187	1									
5	0.971	6.658	–0.094	0.0369	0.0101	–0.0258	1								
6	1.332	1.123	0.1535	–0.1183	–0.1024	–0.0071	–0.0093	1							
7	21.904	29.034	–0.0231	0.0694	–0.0284	–0.0133	–0.0065	–0.0442	1						
8	4.486	19.982	0.0852	–0.0811	–0.0573	0.0206	–0.0031	0.0587	–0.0598	1					
9	4.416	1.063	0.1105	–0.2476	0.0482	–0.0085	–0.0353	0.1014	0.1329	–0.0601	1				
10	1.337	48.215	0.0173	–0.0136	0.0186	0.0029	0.0004	–0.0069	0.0054	–0.0071	0.0079	1			
11	149.882	10677.67	0.0212	–0.0061	–0.0133	0.0036	–0.001	–0.0092	–0.0099	–0.0054	–0.0119	0.0831	1		
12	238.156	3344.20	0.0562	0.003	–0.0226	0.0026	–0.0016	–0.0199	–0.0078	–0.0169	–0.0463	–0.0022	–0.0011	1	
13	135175.5	3448960	0.0897	0.0926	0.0007	0.0161	–0.0008	0.0014	–0.0159	0.0065	–0.006	–0.001	–0.0006	0.0079	1

*1. KEJI score, 2. firm size, 3. firm age, 4. roa, 5. slack, 6. Tobin’s Q, 7. fsts, 8. industry sales growth, 9. industry rivalry, 10. negative social CSR performance feedback, 11. negative historical social CSR performance feedback, 12. positive social CSR performance feedback, 13. positive historical social CSR performance feedback.*

Table 2 shows the results of random effect GLS analysis performed for testing the hypothesis which anticipates the effect of performance feedback on CSR performance. Before examining the hypothesis test results, among the control variables, the variables confirmed to have a statistically significant influence on the CSR performance were firm size, slack, Tobin’s Q, industry sales growth, and industry rivalry.

**TABLE 2:** Random effects GLS model (full sample).

Variables	Model 1	Model 2
Firm size	0.3929***	0.3788***
	(0.0579)	(0.0584)
Firm age	−0.0080	−0.0078^†^
	(0.0045)	(0.0045)
ROA	−0.3290	−0.3647
	(0.7129)	(0.7076)
Slack	−0.3057**	−0.3106**
	(0.1147)	(0.1146)
Tobin’s Q	0.2224*	0.2245*
	(0.1125)	(0.1122)
FSTS	−0.0014	−0.0013
	(0.0031)	(0.0031)
Industry sales growth	0.0107**	0.0113**
	(0.0038)	(0.0038)
Industry rivalry	−0.2141	11.5930*
	(1.4428)	(4.8859)
Negative social CSR performance feedback		0.0010
		(0.0022)
Negative historical CSR performance feedback		0.0001
		(0.0002)
Positive social CSR performance feedback		0.0000**
		(0.0000)
Positive historical CSR performance feedback		0.0000**
		(0.0000)
Industry dummy	Included	Included
Intercept	52.2423***	36.2426***
	(2.3807)	(7.5132)
R-sq	0.191	0.205
Wald χ^2^	171.25***	191.21***
N	1,091	1,091

*Robust standard errors are in parentheses;*

^†^p < 0.10, *p < 0.05, **p < 0.01, ***p < 0.001. Industry dummy variables are included, but not reported to save space.

According to Model 1 in Table 2, firm size had a positive impact on CSR performance (*p* ≤ 0.001). This implies that large-size firms receive most of the social attention, which, in turn, increases stakeholder pressure. Therefore, those firms are highly expected to exhibit socially responsible behavior (Choi et al., 2018; Xu and Zeng, 2020). The corporate value measured as Tobin’s *Q* was found to be a positive effect on the CSR performance (*p* ≤ 0.05). This may attributable to visibility and can be explained as similar to the logic of the effect of the firm size on the CRS performance.

Conversely, firm slack resource was found to have a negative (-) impact on CSR performance (*p* ≤ 0.01). As we measured the slack resource as a debt ratio, a high debt ratio may represent the perception of resource which, in turn, may discourage firms to invest in CSR towing to resource constraints. This result remains consistent with that of Attig et al. (2016), wherein the negative relationship between slack and CSR performance and potential slack calculated as the ratio of total debt to total equity was demonstrated to represent that situation, as the more debt the firm has, the less money they can borrow. Also, the control variables for industry, among industry sales growth had a positive (+) effect on the CSR performance (*p* ≤ 0.01). This result shows that the higher industry sales growth means firms in this industry participate more in CSR.

Hypothesis 1 predicted that negative CSR performance feedback in that CSR performance falling below CSR aspiration level is positively related to CSR performance. Hypothesis 2 predicted that positive CSR performance feedback in that as CSR performance rises above the CSR aspiration level is positively related to CSR performance. Our results in Model 2 of Table 2 showed that both positive social performance and historical performance feedbacks were confirmed to have a statistically significant positive effect on the CSR performance (*p* ≤ 0.01 and *p* ≤ 0.01, respectively). However, both negative historical and social performance feedback are not statistically significant to CSR performance. Therefore, Hypothesis 1 was not supported, and Hypothesis 2 was supported.

### Additional Analysis

We performed additional analysis to confirm our empirical results. The sample of this study includes both manufacturing and non-manufacturing industries because 200 companies with excellent CSR activities selected by the Economic Justice Research Institute were targeted. Considering the claims of previous studies that manufacturing is a core industry in Korea and the influence of CSR in manufacturing is stronger in manufacturing (Chung et al., 2018), an additional analysis was conducted only on manufacturing to confirm the result of the entire sample.

Table 3 shows the results of testing the hypothesis by classifying samples of firms in the manufacturing industry. The results of analyzing only firms in the manufacturing industry showed that both the positive social performance feedback and the historical performance feedback had a positive (+) effect on the CSR performance (*p* ≤ 0.01 and *p* ≤ 0.05, respectively).

**TABLE 3:** Random effects GLS model (manufacturing industry).

Variables	Model 1	Model 2
Firm size	0.4483***	0.4338***
	(0.0648)	(0.0654)
Firm age	−0.0101*	−0.0100*
	(0.0049)	(0.0049)
ROA	−0.1356	−0.1739
	(0.7167)	(0.7114)
Slack	−0.1917	−0.2003
	(0.1255)	(0.1253)
Tobin’s Q	0.2464^†^	0.2440^†^
	(0.1291)	(0.1286)
FSTS	−0.0013	−0.0012
	(0.0034)	(0.0034)
Industry sales growth	0.0108**	0.0116**
	(0.0041)	(0.0041)
Industry rivalry	−0.5260	−0.5180
	(0.9893)	(0.9837)
Negative social CSR performance feedback		0.0011
		(0.0021)
Negative historical CSR performance feedback		0.0001
		(0.0001)
Positive social CSR performance feedback		0.0000**
		(0.0000)
Positive historical CSR performance feedback		0.0000*
		(0.0000)
Industry dummy	Included	Included
Intercept	54.5995***	54.9580***
	(4.35131)	(4.3352)
R-sq	0.1844	0.1993
Wald χ^2^	128.61***	147.13***
N	907	907

*Robust standard errors are in parentheses;*

^†^p < 0.10, *p < 0.05, **p < 0.01, ***p < 0.001. Industry dummy variables are included, but not reported to save space.

## Discussion and Conclusion

Considering the growing social interest in CSR, this study contributes to CSR literature by analyzing the CSR determinants from the BTOF perspective. Based on the perspective of the BTOF and RBV, this study empirically analyzed the CSR performance feedback on CSR performance. To test the hypotheses, we performed GLS regression analysis based on 2012–2019 Korean company data. We found that positive social and historical performance feedback had a positive effect on CSR performance. Our results showed that positive social and historical performance feedback have positive impact on the CSR performance. These results imply that the difference in CSR performance compared to a firm’s CSR aspiration level (positive or negative attainment discrepancy) influences the firm’s strategy or behavior related to CSR according to the performance feedback perspective of BTOF. Especially, if a firm receives positive CSR performance feedback, they increase their efforts in CSR activities to secure authenticity and legitimacy for CSR activities from stakeholders. This includes consumers and can perform more experimental and active CSR activities to strengthen their positive corporate image (Jeong et al., 2018; Vogler and Eisenegger, 2021).

This study provides the following theoretical and empirical contributions to CSR and BTOF research fields. First, this study contributes to CSR literature by applying BTOF discussions to the CSR context by theorizing the performance feedback as the significant determinants to CSR performance and empirically testing the relationship between two variables. Specifically, this study showed that the strategic behaviors that firms choose may vary depending on whether CSR performance is high or low compared to aspiration level. Additionally, we examined both the historical and social aspirations of CSR performance feedback on CSR performance. By doing so, this research empirically confirms both the historical and social aspirations of CSR attainment on CSR performance.

Second, our study expands BTOF literature by examining the CSR aspiration of both historical and social aspiration level and its performance implication. According to a recent study, it is argued that it is not advisable to use a combination of the two variables because historical aspirations and social aspirations are fundamentally different in terms of their characteristics and influence (Deb et al., 2019). The previous studies have not sufficiently addressed the effect of the CSR performance feedback and the difference between CSR aspiration level and actual CSR performance. Thus, we answer a recent call for more research examining the CSR performance feedback research. As Nason et al. (2018) highlighted the discussion on the social performance feedback compared to financial performance feedback is considerably lacking. Attempts to examine how a firm’s strategic behavior changes according to corporate non-financial social performance feedback have been relatively insufficient so far.

Despite the above-mentioned contributions, this study has several limitations as follows. First, as this study examined CSR aspiration level on the CSR performance of Korean firms, generalization in interpreting the results may be difficult. Therefore, future research should investigate whether this study’s results can be generalized to countries other than Korea. Particularly, as the degree of social interest by country in corporate CSR—along with the pressure received by firms—may vary, future research should examine the relationship between CSR aspiration based on the BTOF on CSR performance using a more diverse sample of countries. Second, the sample of this study is 200 companies with excellent CSR activities selected by the Economic Justice Research Institute. We used the sample that matched the KEJI index and firm-level information to those firms selected in the top 200 selected by KEJI, there may be a problem of a sample selection bias (Oh et al., 2019). Third, CSR activities are related to the use of significant resources. The type of slack resource is highly likely to affect CSR results from the BTOF perspective (Greve, 2003). Although this study could not consider the moderating role of these slack resources, analyzing the moderating role of various types of slack resources on this research context in future studies will be promising. Fourth, corporate reputation can be considered the strategic assets held by firms that are a source of a sustainable competitive advantage (Wernerfelt, 1984; Barney, 1991; Roberts and Dowling, 2002; Eberl and Schwaiger, 2005), which, in turn, may have impact on CSR performance. Therefore, future research should examine the impact of corporate reputation on CSR performance. Finally, this study could not consider the impact of corporate governance as a control variable. Hence, we recommend exploring corporate governance as a control variable in future research.

## Data Availability Statement

The raw data supporting the conclusions of this article will be made available by the authors, without undue reservation.

## Author Contributions

J-EL and YY contributed to conception and design of the study, and wrote the first draft of the manuscript. YY organized the database, performed the statistical analysis, and wrote “Research Methods” and “Discussion and Conclusion” sections of the manuscript. J-EL wrote “Introduction” and “Theoretical Background and Hypothesis Development” sections of the manuscript. Both authors contributed to manuscript revision, read, and approved the submitted version.

## Conflict of Interest

The authors declare that the research was conducted in the absence of any commercial or financial relationships that could be construed as a potential conflict of interest.

## Publisher’s Note

All claims expressed in this article are solely those of the authors and do not necessarily represent those of their affiliated organizations, or those of the publisher, the editors and the reviewers. Any product that may be evaluated in this article, or claim that may be made by its manufacturer, is not guaranteed or endorsed by the publisher.
